# Outcomes of modern antiretroviral therapy in obese individuals living with HIV

**DOI:** 10.1093/jac/dkac368

**Published:** 2022-11-02

**Authors:** L Zino, J Stalenhoef, A Colbers, D M Burger

**Affiliations:** Department of Pharmacy and Radboud Institute for Health Sciences, Radboud University Medical Center, Nijmegen, The Netherlands; Department of Internal Medicine, OLVG Hospital, Amsterdam, The Netherlands; Department of Pharmacy and Radboud Institute for Health Sciences, Radboud University Medical Center, Nijmegen, The Netherlands; Department of Pharmacy and Radboud Institute for Health Sciences, Radboud University Medical Center, Nijmegen, The Netherlands

## Abstract

Obesity is a global epidemic and people living with HIV (PLWH) are showing similar obesity trends to those in the general population. Obesity is manifested by several physiological features that can alter volume of distribution, elimination and metabolism of various medications including ART. Some drugs are increasingly prone to pharmacokinetic alteration during obesity depending on their physicochemical properties and clearance mechanism. These considerations raise concerns of hampered efficacy, development of resistance or increased toxicity of ART in PLWH. Here, we summarize available literature on the exposure and antiviral outcomes of currently available antiretroviral drugs in the context of obesity and provide a panel of recommendations for the clinical management and follow-up in this growing patient population.

## Introduction

Prevalence of obesity (BMI ≥ 30 kg/m^2^) among people living with HIV (PLWH) is hitting high. Besides being a global phenomenon, progressive weight gain is an apparent feature in people initiating ART; particularly those containing integrase inhibitors and tenofovir alafenamide fumarate, with no clear causative mechanism.^1,2^ Obesity is linked to several physiological alterations that affect drug pharmacokinetics (PK).^3^ Obesity is characterized by an accumulation of fat tissue, increased lean mass and plasma volume, which can increase volume of distribution for several medications. Elimination is also affected by the increased organ size, including liver and kidneys, and the accelerated glomerular filtration. Other physiology parameters including increased cardiac output and altered splanchnic and systemic blood flow are also observed in obesity.^4–7^ Moreover, obesity can alter drug metabolism via its impact on cytochrome P450 (CYP) isoforms. Activity of CYP3A4, a major drug-metabolizing enzyme, is reduced by 35% for several probes in obese individuals compared with their lean controls.^8,9^ A recent systematic review concluded that activity of CYP2C9, CYP1A2 and CYP3A4/5 is reduced in obese individuals, while CYP2E1 activity is enhanced.^10^ Furthermore, the chronic inflammation status linked to obesity may contribute to substantial alterations in drug-metabolizing enzymes and transporters,^11^ which adds another level of PK variability in subjects with obesity. Depending on their physicochemical properties and clearance site, some antiretrovirals are expected to have altered PK during obesity, which requires further investigation.

As important as the effect on the PK parameters, obesity contributes to several comorbidities in PLWH including cardiovascular disease, diabetes and hypertension.^12,13^ Management of these multiple comorbidities requires mostly polypharmacy, which increases the risk of developing drug–drug interactions with ART.

Together, these considerations raise concerns of lowered efficacy, development of resistance and/or increased toxicity of ART in PLWH. To date, few studies have reported outcomes of ART in the cohort of obese or morbidly obese (BMI > 40 kg/m^2^, referred as Class 3 obesity)^14^ PLWH. HIV treatment guidelines are also lacking for these populations. Here, we summarize the current knowledge on the influence of obesity on the plasma concentrations and activity of ART agents from published clinical trials and case reports up to March 2022.

## Methods

We conducted a literature search using PubMed and Google Scholar between January and April 2022. Search terms that were used to retrieve data included ‘obesity pharmacokinetics’ in combination with ‘antiretroviral, anti-HIV therapy, ART or anti-HIV medications’. Backward reference searching from primarily retrieved literature also contributed to the total number of retrieved reports and conference abstracts.

### NRTIs

Many NRTIs are cleared by the kidneys, thus could be affected by accelerated renal clearance in obese individuals.^15^ Tenofovir disoproxil fumarate is a prodrug that is hydrolysed quickly to tenofovir in the plasma. Tenofovir disoproxil fumarate shows efficient permeability through lipid membranes, increased oral absorption and distribution of the active metabolite across circulating lymphoid cells as well as plasma compared with tenofovir in macaques.^16^ A study on four subjects on a tenofovir disoproxil/emtricitabine-containing regimen showed that obesity [mean (±SD) BMI of 42.7 (± 4.3) kg/m^2^] had no impact on time to maximum concentration (*T*_max_ = 1–1.5 h) or trough plasma concentrations (*C*_24_ = 47 ± 15 ng/mL) of tenofovir in these four subjects compared with population levels from literature (*C*_24_ = 15–180 ng/mL). Subjects in this analysis were planned to undergo bariatric surgery and were all virally supressed before the surgery.^17^

A large comparable study reported that among current NRTIs, median tenofovir concentrations at 12 h (*C*_12_) were significantly lower in obese subjects (*n* = 151, BMI > 30 kg/m^2^) compared with non-obese controls (*n* = 117, BMI = 19–25 kg/m^2^): 66 versus 86 ng/mL, with no difference in antiviral activity nor time to viral suppression.^18^ Notably, the difference in plasma levels was significant (−23%) only when tenofovir disoproxil fumarate 300 mg once daily was administered within regimens containing two NRTIs and one NNRTI, rather than two NRTIs plus one PI.^18^ A probable mechanism is the PI’s influence on renal clearance, which can decrease tenofovir clearance and retain its plasma levels.^19^ In both studies, obesity did not lower tenofovir trough concentrations below the minimum population levels. Data on the novel tenofovir prodrug, tenofovir alafenamide fumarate, in the context of (morbid) obesity are scarce. A PK study investigated the influence of genetic polymorphism and non-genetic factors in Chinese PLWH and reported a positive correlation between the AUC_0–*t*_ of tenofovir alafenamide fumarate and BMI. However, this correlation was proven to be non-significant in multivariate analysis.^20^ Nevertheless, the study enrolled subjects from a restricted range of BMI (19–26 kg/m^2^) and, therefore, needs to replicated in a wider range of BMI categories including BMI >30 kg/m^2^.

In the study of Madelain and colleagues,^18^ concentrations at 12 h (*C*_12_) for emtricitabine (*n* = 234), abacavir (*n* = 69) and lamivudine (*n* = 76) were similar between obese and non-obese individuals with no signs of suboptimal trough concentrations or drug toxicity (Table 1).

**Table 1.** Key PK changes of modern ART in individuals with obesity

Drug	Dose	PK outcomes	PK measures in obesity^a^ (median ± range)	Population/control (ng/mL)	prevalence of *C*_trough_ < effective concentration	Recommendation in obesity
NRTIs						
Abacavir	600 mg once daily^18^	*C*_12_↔	*C*_12_ = 58 ng/mL	*C*_12_ = 47 ng/mL	similar	Use standard dose
Emtricitabine	200 mg once daily^18^	*C*_12_↔	*C*_12_ = 180 ng/mL	*C*_12_ = 201 ng/mL	similar	Use standard dose
Lamivudine	300 mg once daily^18^	*C*_12_↔	*C*_12_ = 181 ng/mL	*C*_12_ = 200 ng/mL	similar	Use standard dose
Tenofovir disoproxil fumarate	300 mg once daily^17^	*T*_max_ ↔ *C*_max_ ↔ *C*_24_ ↔ AUC ↔ CL ↔	*T*_max_ = 1–1.5 h^b^*C*_max_ = 263 ± 79 ng/mL^b^*C*_24 _= 47 ± 15 ng/mL^b^AUC = 2346 ± 643 ng·h/mL^b^CL = 112 ± 37 L/h^b^	—	similar	Use under therapeutic drug monitoring during obesity if administered in two NRTIs and one NNRTI regimen.
	300 mg once daily^18^	*C*_12_ ↓ 23%	*C*_12_ = 66 ± 18 ng/mL	*C*_12_ = 86 ± 32 ng/mL	—	
NNRTIs						
Efavirenz	Several once daily doses, *n* = 1^15^; 600 mg 1200 mg 1800 mg	—	806 ng/mL^b^ at 18 h 1422 ng/mL^b^ at 12 h 2030 ng/mL^b^ at 11 h	—	Subtherapeutic at 600 mg once daily dose^c^	Use under therapeutic drug monitoring during morbid obesity and avoid any exposure-lowering factors
	600 mg once daily^18^	*C*_12_ ↓ 26%	*C*_12_ = 1498 ng/mL (1091–2292)	*C*_12_ = 2034 ng/mL (1566–3181)	19% of *n* below efficacy threshold versus 0% in non-obese population^c^	
Etravirine	400 mg once daily or 200 mg twice daily^18^	*C*_12_↔	*C*_12_ = 808 ng/mL	*C*_12_ = 674 ng/mL	similar	Use standard dose
Nevirapine	200 mg twice daily^18^	*C*_12_↔	*C*_12_ = 4681 ng/mL	*C*_12_ = 4624 ng/mL	similar	Use standard dose
PIs						
Atazanavir	300 mg plus ritonavir 100 mg once daily^3,18^	*C*_24_↔ *C*_24_↔	*C*_24_ = 754 ng/mL *C*_24_ = 999 ± 957 ng/mL^b,d^	*C*_24_ = 694 ng/mL *C*_24_ = 780 ± 740	similar	Use standard dose
Darunavir	800 mg plus ritonavir 100 mg once daily^3,18^	*C*_24_↔ *C*_24_↔	*C*_24_ = 3287.7 ± 223.1 ng/mL^b^ *C*_24_ = 1481 ng/mL	*C*_24_ = 2077.6 ± 1082 ng/mL *C*_24_ = 1266 ng/mL	Similar	Use standard dose
	600 mg plus ritonavir 100 mg twice daily^18^	*C*_12_↔	*C*_12_ = 2982 ng/mL	*C*_12_ = 2818 ng/mL	Similar	No dose change required
Lopinavir	400 mg plus ritonavir 100 mg twice daily^18^	*C*_12_ ↓28%	*C*_12_ = 4595 ng/mL	*C*_12_ = 6420 ng/mL	24.4% of *n* below efficacy threshold versus 0% in non-obese population^e^	Use under therapeutic drug monitoring during obesity and avoid any exposure-lowering factors
Ritonavir	100 mg once daily^18^	*C*_12_ ↓57%	*C*_24_ = 69 (33–115) ng/mL	*C*_24 _= 162 (50–303) ng/mL	—	
	100 mg twice daily^18^	*C*_12_ ↓69%	*C*_12_ = 79 (40–123) ng/mL	*C*_12_ = 256 (150–370) ng/mL	—	
INSTIs						
Bictegravir	50 mg once daily (modelling only)^21^	AUC ↓11% *C*_max_ ↓15%	AUC = 64 343.5^b^ ng·h/mL *C*_max _= 3485.7^b^ ng/mL	AUC = 72 689.5 ng·h/mL *C*_max _= 4095.6 ng/mL	—	Use standard dose
Dolutegravir	50 mg once daily^21^	AUC ↓5% *C*_max_ ↓12%	AUC = 43 415.8^b^ ng·h/mL *C*_max _= 2884.3^b^ ng/mL	AUC = 45 445.8 ng·h/mL *C*_max _= 3316.9 ng/mL	—	Use standard dose
Raltegravir	400 mg twice daily^18^	*C*_12_ ↓44%	*C*_12_ = 120 ng/mL	*C*_12_ = 215 ng/mL	17% of *n* below efficacy threshold versus 0% in non-obese population	Use standard dos

AUC; area under the concentration-time curve; *C*_12_, concentration at 12 h or trough concentration for twice daily dosing; *C*_24_, concentration at 24 h or trough concentration for once daily dosing; *C*_trough_, concentration at the end of the dosing interval; CL, clearance; T_max_, time to maximum concentration.

a Obesity is defined as BMI ≥ 30 kg/m^2^.

b Concentrations are represented by mean (±SD).

c Efficacy threshold of efavirenz was set at 1000 ng/mL. The current recommendation for efavirenz mid-dose threshold is 700 ng/mL.^22^

d This concentration is reported in people with BMI ≥ 25 kg/m^2^ rather than  ≥ 30 kg/m^2^.

e Clinical efficacy threshold for lopinavir is 3000 ng/mL.^23^

As such, no specific recommendations exist for most NRTIs in the obese population. Monitoring tenofovir plasma concentrations may be considered if administered in regimens containing two NRTIs and one NNRTI in patients with suboptimal viral control. Data are lacking for tenofovir concentrations when combined with integrase inhibitors.

### NNRTIs

Theoretically, efavirenz poses a risk of increased variability of antiretroviral PK in subjects with obesity. Efavirenz has a high lipophilicity (logP 4.6) and a large affinity for fat, which is reflected by 100-fold higher concentrations in adipose tissue compared with plasma.^24^ Moreover, multivariable models predicted 10% lower plasma concentrations of efavirenz for each additional 10 kg of weight.^25^ A case report of an ART-naive subject with extreme obesity (BMI > 66 kg/m^2^) detected a subtherapeutic concentration of efavirenz (806 ng/mL) at 18 h on a standard dose of 600 mg once daily (plus tenofovir disoproxil/emtricitabine).^15^ The dose was doubled to 1200 mg once daily and the efavirenz level at 12 h was 1855 ng/mL,^15^ which falls within the therapeutic range (1000–4000 ng/mL).^26^ However, the dose was tripled temporarily for 4 weeks (1800 mg once daily) due to persisting low viraemia (58 copies/mL) with circulating plasma levels below the safety threshold (2030 ng/mL). Using physiologically based PK modelling (PBPK), the authors simulated a weight-dependent dosing scheme for efavirenz in virtual obese populations and suggested a range of 600–1800 mg of daily efavirenz to attain the therapeutic range in several weight groups.^15^ However, NRTI levels, including tenofovir, were never measured in this patient. As mentioned above, subtherapeutic plasma concentrations of the NRTI backbone, particularly tenofovir, is probable in obese patients within regimens containing two NRTIs and one NNRTI. Thus, it may have contributed to the late viral suppression when efavirenz levels were therapeutic during 1200 mg efavirenz dosing. Generally, data from a single case report should be interpreted with caution, as the final drug concentrations might be influenced by many other factors, such as the patient’s adherence and unknown drug–drug interactions, making it challenging to distinguish the effect of obesity solely on the drug concentrations.

A study with a large ART-naive HIV cohort (*n* = 19 968) investigated the effect of several weight bands (<55 to >95 kg) on efavirenz antiviral and immunological response and found no statistical difference between all weight groups.^27^ The study concluded that standard 600 mg efavirenz dose is sufficient in patients across various weight ranges. However, the study stratified subjects by weight instead of BMI, which is a better estimation for lean/fat ratio. Nevertheless, efavirenz exposure is unlikely to differ in obesity since investigators did not detect any significant difference in heavier subjects who weighed >95 kg (*n* = 854) up to 1 year of follow-up.^27^

Madelain *et al.*^18^ confirmed similar efficacy patterns for efavirenz among obese (*n* = 78, BMI > 30 kg/m^2^) and non-obese controls (*n* = 51, BMI = 19–25 kg/m^2^), with obese individuals being more likely to have *C*_12_ below the efficacy threshold (19% obese versus 0% controls). Median *C*_12_ concentrations were significantly lower in obese subjects (1498 ng/mL) compared with the lean controls (2034 ng/mL). Notably, the study set efficacy threshold for efavirenz was 1000 ng/mL, which was later reduced to 700 ng/mL.^22^

Together, these studies highlight the potential of subtherapeutic exposure of efavirenz, but not necessarily treatment failure in obese PLWH. In the case of persistent viraemia, suspected virological failure, or when an exposure-lowering factor exists, performing therapeutic drug monitoring for efavirenz is recommended in people with obesity. Clinical and trough plasma concentrations data on other NNRTI drugs from the same study does not appear to be of concern for patients with obesity,^18^ including etravirine and nevirapine (Table 1). No data exist for the exposure or virological efficacy of rilpivirine or the novel doravirine in subjects with obesity.

### PIs

CYP3A4 is the major enzyme contributing to the metabolism of PIs. CYP3A4 activity decreases in obese individuals,^10^ and tends to increase after losing weight due to weight-loss surgery.^28^ Increased free circulating levels of PIs due to decreased metabolic clearance are expected in obese individuals. On the other hand, drug metabolism is positively correlated with hepatic blood flow and cardiac output, which are greater during obesity.^29^ This may explain why, despite the lowered CYP3A4 activity, increased weight has a minimal effect on some PIs, such as darunavir and atazanavir.^3,18^

Trough concentrations of three ritonavir-boosted PIs, lopinavir (twice daily), atazanavir (once daily) and darunavir (once and twice daily) in obese people (BMI > 30 kg/m^2^) were not above those of their matched controls (BMI = 19–25 kg/m^2^) in a comparative study (Table 1).^18^ Instead, levels of lopinavir/ritonavir 400/100 mg twice daily at 12 h were 28% lower in obese people (*n* = 45) compared with controls (*n* = 33) with 24% incidence of *C*_12_ below the efficacy threshold (3000 ng/mL)^23^ in obese versus lean controls.^18^ Lower plasma concentrations in obese people are unforeseen because lopinavir has a shorter half-life of 5–6 h and fat accumulation characteristics were never reported in the PI class. Thus, it is challenging to confirm the adequacy of lopinavir trough concentrations in the obese population, which should warrant further research.

By contrast, trough concentrations of boosted darunavir were slightly, but not significantly, higher in obese (*n*= 70, BMI > 30 kg/m^2^) and overweight (*n* = 7, BMI > 25 kg/m^2^) subjects compared with non-obese controls (*n* = 52 and 23, BMI < 25 kg/m^2^) for both once- and twice-daily dosing regimens.^3,18^ Incidence of having *C*_12_ or *C*_24_ under the efficacy threshold (protein-binding adjusted EC_50_ = 55 ng/mL) was not higher in subjects with obesity compared with the non-obese controls.^18^ When incorporating body composition measurements, boosted darunavir 800 mg once-daily concentrations were positively correlated with higher fat mass (r = 0.3, *P* = 0.02), especially in subjects from sub-Saharan origin.^3^ Yet, data on darunavir indicate a negligible effect of weight on trough concentrations with no evidence of clinical relevance. The same goes for atazanavir/ritonavir 300/100 mg once daily, which showed similar trough concentrations across obese/overweight (*n* = 67 and 20) and lean controls (*n* = 29 and 23).^3,18^ There was no increased incidence of having *C*_trough_ out of safety and efficacy boundaries in subjects with obesity.^18^

Median (range) trough levels of the ritonavir booster given as 100 mg once daily or 100 mg twice daily were significantly lower in obese individuals: 69 ng/mL (33–115) and 79 (40–123), compared with 162 ng/mL (50–303) and 256 (150–370) in controls, respectively.^18^ Currently, data on cobicistat boosted PIs are lacking.

The above-mentioned studies suggest that among PIs, ritonavir-boosted lopinavir poses a potential risk of decreased trough concentrations in obese individuals. Hence, one can conceivably hypothesize that lopinavir is less suitable for switching patients with obesity when other alternatives exist, and that therapeutic drug monitoring is recommended in subjects on lopinavir treatment with poor viral control.

### Integrase strand transfer inhibitors (INSTIs)

Emergent obesity is more profound in PLWH initiating INSTIs compared with other ART classes.^30^ Fortunately, the limited existing data do not indicate any negative impact of obesity on INSTI PK. Trough concentrations of raltegravir 400 mg twice daily (given in combination with ritonavir-boosted atazanavir) were lower in obese (*n* = 28, BMI > 30 kg/m^2^) compared with non-obese (*n* = 24, BMI = 19–25 kg/m^2^) individuals with *C*_12_ at 120 ng/mL and 215 ng/mL, respectively. However, the authors indicated that calculating the significance of the difference in concentrations was difficult due to the small number of subjects in both groups and the high variability in the measured plasma concentrations.^18^ Incidence of *C*_12_ below the efficacy threshold was higher in obese people (17% versus 0%) but with no deleterious effect for the virological control up to 1 year post assessment.^18^ Absorption of raltegravir is dependent on high gastric acidity.^31^ Hence, the authors speculated that since ∼50% of obese individuals develop gastro-oesophageal reflux disease,^32^ raltegravir absorption might be reduced in these patients due to the concomitant use of acid-reducing agents. As mentioned above, the high variability in plasma levels among this small sample of obese patients (*n* < 30), makes it difficult to draw definitive conclusions.

A recent analysis showed that neither observed nor PBPK-predicted PK parameters of dolutegravir denote a clinically relevant change in AUC between obese (43 415 ng·h/mL, BMI = 30–40 kg/m^2^) and non-obese PLWH (45 445 ng·h/mL, BMI = 18.5–30 kg/m^2^). Also for bictegravir, modest and not clinically relevant lower steady-state concentrations were demonstrated by PBPK predictions that are verified by clinical observations.^21^

Due to its recent approval, data on long-acting (LA) cabotegravir during obesity is limited to drug development trials. PK analysis from FLAIR and ATLAS, Phase 3 trials on cabotegravir and LA rilpivirine, showed that plasma concentrations of cabotegravir were lower in subjects with BMI ≥ 30 kg/m^2^ at the initial doses.^33^ Investigators attributed the decreased concentrations of cabotegravir to the delayed absorption across the adipose tissue. Importantly, plasma concentrations of cabotegravir were comparable to those measured in non-obese by Week 48. Plasma concentrations of rilpivirine were similar across BMI categories.^33^

As INSTIs are becoming first-line therapy in many settings, further research is warranted for INSTIs where data in obese PLWH are non-comprehensive or lacking, as in the case of elvitegravir.

## Conclusions

So far, most antiretroviral drugs appear not to be at risk of treatment failure or toxic exposure in PLWH and obesity. Signs of caution exist for tenofovir disoproxil fumarate and lopinavir in obese individuals, as well as efavirenz in extremely obese subjects, which encourages therapeutic drug monitoring in these settings. Additionally, management of the drug–drug interaction between acid-reducing agents and raltegravir should be considered in PLWH and obesity. Although the studies for several ART and dosing regimens showed minimal clinical relevance in obesity, knowledge gaps exist for several other antiretroviral drugs. As obesity incidence increases in PLWH, we recommend that trough concentrations and AUC of newer antiretroviral drugs, as well as the impact of their physicochemical properties, should be investigated in the setting of obesity.
